# Supplementary material for: Visuomotor Adaptation Modulates the Clustering of Sleep Spindles Into Trains
Source: Front Neurosci. 2022 Mar 16;16:803387. doi: 10.3389/fnins.2022.803387 (PMC8966394; doi:10.3389/fnins.2022.803387)
Supplement: Supplementary file 1 [file Data_Sheet_1.docx]

**Supplementary Figures**

**
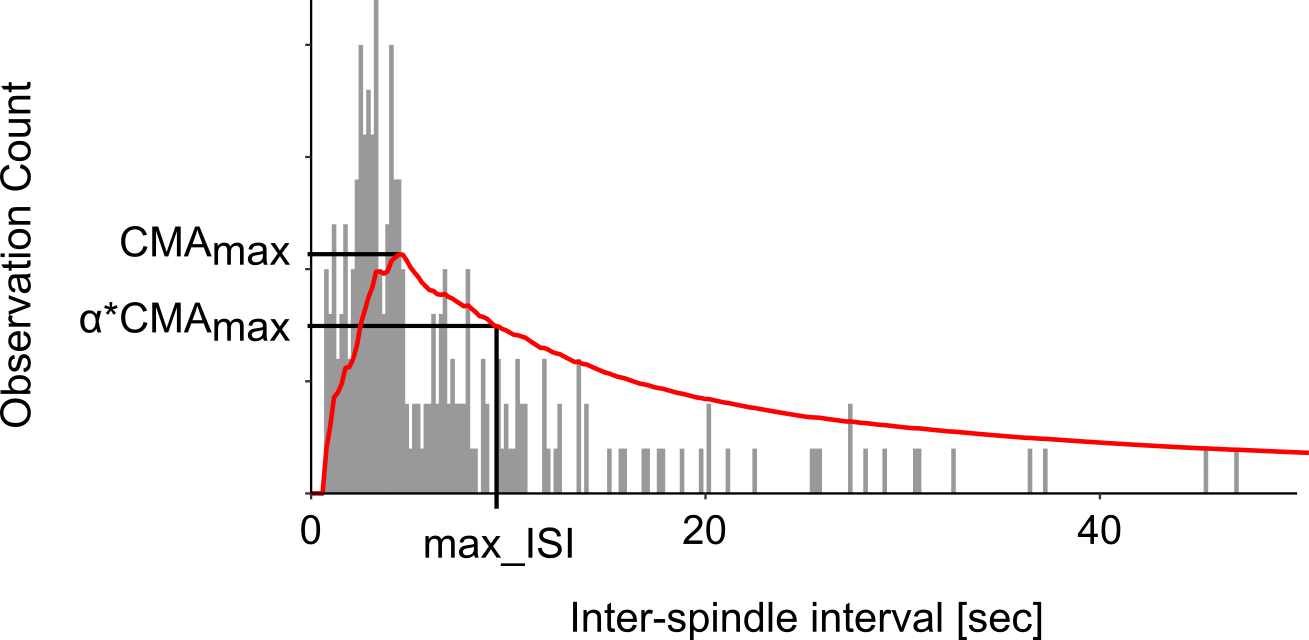
**

**Supplementary Figure 1. Identification of the max_ISI threshold using the CMA curve.** Shown is the histogram of the inter-spindle intervals (ISI) for an example subject (gray bars), and the Cumulative Moving Average (CMA) curve computed for this histogram (red curve). Based on the skewness of the CMA curve, a parameter ɑ (*ɑ=1* if *Skewness < 1*; *ɑ=0.7* if *1 ≤ Skewness < 4*; *ɑ=0.5* if *4 ≤ Skewness < 9*; and *ɑ=0.3* if *Skewness ≥ 9)* is determined and used to scale the maximum of the CMA curve (CMA_max_). The CMA_max_ and the CMA_max_ multiplied by ɑ (ɑ*CMA_max_) are highlighted on the CMA curve. Note that the max_ISI corresponds to the ISI where the CMA curve falls to ɑ*CMA_max_. This max_ISI can be used as the threshold for spindle trains detection.

**Supplementary Tables**

|  | **Familiarization** | **VMA** | **CTL** | **LMM** |
| --- | --- | --- | --- | --- |
| **Measure** | Mean (SE) | Mean (SE) | Mean (SE) | F (p) |
| **Sleep latency** | 18.20 (4.95) | 16.05 (3.40) | 18.40 (4.35) | 0.34 (0.71) |
| **REM latency** | 132.60 (20.81) | 123.85 (15.66) | 105.00 (9.88) | 1.09 (0.35) |
| **NREM1** | 43.35 (7.43) | 53.35 (5.14) | 53.30 (9.69) | 1.17 (0.33) |
| **NREM2** | 157.30 (17.68) | 150.15 (14.70) | 157.25 (11.48) | 0.19 (0.82) |
| **NREM3** | 100.25 (12.27) | 105.90 (7.43) | 100.15 (7.45) | 0.15 (0.86) |
| **REM** | 75.50 (10.92) | 77.45 (8.48) | 75.40 (9.23) | 0.03 (0.97) |
| **WASO** | 42.95 (19.93) | 29.50 (6.40) | 33.10 (8.95) | 0.41 (0.67) |
| **Total Wake Time** | 80.35 (24.50) | 58.75 (14.48) | 54.80 (9.67) | 1.01 (0.38) |
| **Sleep Efficiency** | 81.90 (5.27) | 86.57 (3.11) | 87.36 (2.19) | 1.01 (0.38) |
| **Total Sleep Time** | 376.40 (23.97) | 386.85 (12.76) | 386.10 (8.91) | 0.18 (0.83) |

**Supplementary Table 1. Sleep Architecture.** Shown are the mean and standard error (SE) corresponding to the sleep measures listed in the first column, for the familiarization, VMA and CTL sessions. LMM’s statistics and p values obtained from comparing the three conditions are depicted in the last column. Measures are depicted in minutes except for Sleep Efficiency (% of total sleep time relative to the time interval between lights-off and lights-on). WASO: wake after sleep onset.

|  | **Duration (ms)** | | | | | | **Amplitude (uV)** | | | | | |
| --- | --- | --- | --- | --- | --- | --- | --- | --- | --- | --- | --- | --- |
| Number of sindles in Train | Isolated Spindle | | First Spindle | | Last Spindle | | Isolated Spindle | | First Spindle | | Last Spindle | |
|  | *Mean* | *SE* | *Mean* | *SE* | *Mean* | *SE* | *Mean* | *SE* | *Mean* | *SE* | *Mean* | *SE* |
| Isolated | 887.0 | 33.5 |  |  |  |  | 52.2 | 3.9 |  |  |  |  |
| 2 |  |  | 890.2 | 31.4 | 940.9 | 35.1 |  |  | 52.5 | 4.1 | 53.1 | 4.0 |
| 3 |  |  | 937.6 | 31.4 | 959.6 | 36.3 |  |  | 52.9 | 4.1 | 55.7 | 4.5 |
| 4 |  |  | 932.2 | 22.9 | 985.3 | 35.6 |  |  | 54.1 | 4.8 | 56.6 | 4.0 |
| 5 |  |  | 987.1 | 26.9 | 1025.7 | 42.0 |  |  | 55.9 | 4.9 | 56.3 | 5.4 |
| 6 |  |  | 1030.2 | 28.9 | 1061.2 | 23.8 |  |  | 56.1 | 3.6 | 55.9 | 3.5 |

**Supplementary Table 2.** Shown are the mean and standard error (SE) corresponding to the duration and amplitude of isolated spindles, and that of the first and last spindles in a train.
